# Validation of the Enzyme-Linked ImmunoSpot Analytic Method for the Detection of Human IFN-γ from Peripheral Blood Mononuclear Cells in Response to the SARS-CoV-2 Spike Protein

**DOI:** 10.3390/biom14101286

**Published:** 2024-10-11

**Authors:** Laura E. Carreto-Binaghi, Milton Nieto-Ponce, Andrea Palencia-Reyes, Rodolfo L. Chávez-Domínguez, Jessica Blancas-Zaragoza, Pablo Franco-Mendoza, Montserrat A. García-Ramos, Claudia I. Hernández-Lázaro, Martha Torres, Claudia Carranza

**Affiliations:** 1 Laboratorio de Inmunobiología de la Tuberculosis, Instituto Nacional de Enfermedades Respiratorias (INER) Ismael Cosío Villegas, Mexico City 14080, Mexico; lcarreto-binaghi@som.umary-land.edu (L.E.C.-B.); milton.nieto@iner.gob.mx (M.N.-P.); andypal93@gmail.com (A.P.-R.); rodolfo_chvz@comunidad.unam.mx (R.L.C.-D.); jessica.blancas@iner.gob.mx (J.B.-Z.); mendoza_1496@hotmail.com (P.F.-M.); montsabi27@gmail.com (M.A.G.-R.); 2 Laboratorio Clinico, Instituto Nacional de Enfermedades Respiratorias (INER) “Ismael Cosío Villegas”, Mexico City 14080, Mexico; c.ivett.hdez@gmail.com

**Keywords:** validation, Elispot, immune response, SARS-CoV2, IFN-γ

## Abstract

COVID-19 vaccine evaluations are mainly focused on antibody analyses, but there is growing interest in measuring the cellular immune responses from the researchers evaluating these vaccines. The cellular responses to several COVID-19 vaccines have been studied using the enzyme-linked immunospot (ELISPOT) assay for IFN-γ. However, the ELISPOT assay is no longer used only for research purpose and so the performance of this assay must be validated. Since the bioanalytical validation of ELISPOT-IFN-γ is essential for evaluating the method’s effectiveness and establishing confidence in a vaccine’s immunogenicity, the present work validates the ELISPOT-IFN-γ assay’s performance in determining the frequency of IFN-γ-producing cells after stimulation with the SARS-CoV-2 spike protein. The validation was performed in peripheral blood mononuclear cells from volunteers immunized with anti-COVID-19 vaccines. According to the findings, the LOD was 17 SFU and the LLOQ was 22 SFU, which makes the method highly sensitive and suitable for evaluating low levels of cellular responses. The procedure’s accuracy is confirmed by the correlation coefficients for the spike protein and anti-CD3^+^, being 0.98 and 0.95, respectively. The repeatability and intermediate precision tests were confirmed to be reliable by obtaining a coefficient of variation of ≤25%. The results obtained in this validation enable the assay to be employed for studying antigen-specific cells and evaluating cellular responses to vaccines.

## 1. Introduction

For the first time in history, different anti-COVID vaccine platforms were established in record time, including vaccines composed of viral mRNA or DNA, protein subunits, attenuated or inactivated viruses, viral vectors, and virus-like particles [1]. One of the main target epitopes is the SARS-CoV-2 spike protein, a surface homotrimer built from two subunits (S1 and S2), which binds to the angiotensin-converting enzyme 2 (ACE2) receptor in human cells and is critical in viral entry and replication during the infection [2,3].

In general, an important caveat in the evaluation of viral vaccines, including COVID-19, is the lack of an appropriate immune correlate of protection, a defined biomarker that would indicate vaccine-induced protection from natural infection [4]. Neutralizing antibodies are generally recommended as the primary correlate of protection [5]; they prevent infection and/or severe disease, and the methodology used for their detection might be easily reproduced in different settings to facilitate comparisons between the data collected in clinical trials using diverse vaccine platforms [6]. Most SARS-CoV-2 vaccines induce specific neutralizing antibodies against the spike protein, therefore blocking its interaction with the ACE2 receptor and preventing the virus from entering into the cell [7]. However, immune protection against viral infections goes beyond the production of antibodies. Cell-mediated immunity largely contributes to long-term responses to viruses, mainly through the recognition of viral proteins for cytokine secretion [8,9,10].

In the context of vaccines, the evaluation of memory T cell responses might be performed by distinct assays, such as proliferation, differentiation towards specific subsets, and the production and release of cytokines upon stimulation with specific antigens [11,12]. SARS-CoV-2 vaccines predominantly induce Th1 responses, characterized by the release of pro-inflammatory cytokines such as tumor necrosis factor alpha (TNFα), interleukin 2 (IL-2), and interferon gamma (IFNγ) [13]. The production and release of IFNγ by effector memory CD4+ and CD8+ T cells is key for the immunoregulatory and antiviral functions of host defense. In this regard, the IFNγ enzyme-linked immunospot assay (ELISPOT-IFNγ) has been routinely used to evaluate cellular responses to various vaccine candidates [14], including those against human immunodeficiency virus [15,16] and SARS-CoV-2 [17,18].

For this purpose, the study of the immune response to specific antigens in clinical trials requires the validation of the technique to assert the reliability of the results in ex vivo tests. However, validation is generally performed for quantitative biomarker detection assays [19], and scarcely for semi-quantitative techniques evaluating cellular responses, such as ELISPOT-IFNγ.

Validation of ELISPOT-IFNγ assay is particularly relevant for assessing the efficacy of the method and its correlation with additional tests for vaccine immunogenicity. Additionally, full compliance with Good Laboratory Practices must always be ensured. To validate a bioanalytical assay, several parameters should be evaluated according to the intended application of the technique and the biological limits of the assay [19].

The upper and lower limits of quantification (LLOQ) represent the range in which the analyte might be measured with accuracy and precision, whereas the limit of detection (LOD) is the minimum concentration of analyte that produces a significantly different signal to the background [20]. Specificity is the ability of the test to identify the analyte from other components and accuracy is the highest approximation of the results with the reference values.

The precision profile of the method is depicted by the coefficient of variation (CV), which relies on repeatability (intra-assay precision), reproducibility (inter-assay or cross validation), and intermediate precision (non-significant changes in the assay conditions) [21]. Depending on the type of assay employed, either dilution linearity or parallelism will determine its accuracy on multiple dilutions of the study sample. Finally, the robustness of the assay indicates the overall reliability of the obtained results regardless of predetermined changes in the procedure.

In the present work we describe the validation of the ELISPOT-IFNγ technique, an ex vivo assay intended to assess the frequency of IFNγ-producing cells after stimulation with the SARS-CoV-2 spike protein, primarily focused on evaluating vaccine-induced T cell-mediated immunity.

## 2. Materials and Methods

### 2.1. Sample Collection

Peripheral blood samples were obtained by venipuncture from healthy donors with an age range of 30–50 years with a previous SARS-CoV-2 infection and complete vaccination with the COVID-19 mRNA vaccine (Pfizer, New York, NY, USA). At the time of venipuncture, the donors presented no symptoms of acute SARS-CoV-2 infection. Considering that specific T cell responses might develop in a volunteer-dependent way, before performing the validation assay, we tested the ELISPOT-IFNγ assay on 10 healthy volunteers. Volunteers with a prior SARS-CoV-2 vaccination and previously confirmed SARS-CoV-2 infection were chosen to ensure that their T cells would show a response to the recombinant Spike S1 protein of SARS-CoV-2 (RayBiotech, Peachtree Corner, GA, USA. The participants were recruited at the National Institute of Respiratory Diseases (INER) in Mexico City. The Institutional Ethics and Research Committee approved this study, and all participants provided written informed consent, per the Declaration of Helsinki.

### 2.2. Isolation and Freezing of Peripheral Blood Mononuclear Cells (PBMCs)

For the validation, we selected the four volunteers with the best responses, whose data are presented in this manuscript. Within the first 2 h after blood collection, PBMCs were isolated as previously described [22]. In brief, whole blood was diluted at a 1:1 ratio with RPMI-1640 medium (Lonza, Walkersville, MD, USA) supplemented with L-glutamine (Lonza, Walkersville, MD, USA) and gentamicin (Gibco, Waltham, MD, USA). The PBMCs were purified using Lymphoprep (Serumwerk, Bernburg, Germany) through a density gradient by centrifugation. The viability of the PBMC always exceeded 98% and was evaluated by manual counting using trypan blue (Sigma, Burlington, MA, USA). Then, the cells were frozen at a concentration of 10 × 10^6^ cells/mL in fetal bovine serum (FBS) (Gibco, Waltham, MA, USA) with 10% of dimethyl sulfoxide (Sigma, Burlington, MA, USA). The vials were placed in a freezing container (Nalgene, Rochester, NY, USA) overnight at −80 °C and then transferred to their definitive storage location at −80 °C until used.

### 2.3. Thawing of PBMC

The PBMCs were thawed using a water bath (Thermo Scientific, Winchester, VA, USA) at 37 °C. They were slowly diluted in 5 mL of OPTmizer-CTS medium (Gibco, Waltham, MA, USA) supplemented with L-glutamine (Lonza, Walkersville, MD, USA) and gentamicin (Gibco, Waltham, MD, USA), which was previously warmed to 37 °C. Following thawing, the PBMCs were centrifuged at 316 rcf for 10 min at room temperature (RT) and resuspended in 1 mL of medium for manual counting using trypan blue (Sigma, Burlington, MA, USA). The cell viability was greater than 98% and their recovery always exceeded 85%. The PBMCs suspension was adjusted to a final concentration of 2 × 10^6^ cells/mL with supplemented OPTmizer-CTS medium (Gibco, Waltham, MA, USA).

### 2.4. Preparation of Stimuli

Stimuli were prepared before starting the assay. Recombinant Spike S1 protein was employed as the target stimulus at a concentration of 5 μg/mL in supplemented OPTmizer-CTS (Gibco, Waltham, MA, USA) medium and Phytohemagglutinin (PHA) (Sigma, Burlington, MA, USA) was used as a positive control at a concentration of 5 μg/mL. This stimulus was tested since it is widely employed to stimulate PBMCs in assays such as ELISAs that quantify IFNγ production [23].

The optimal concentration of the stimuli was determined in previous titration experiments. Additionally, the human anti-CD3^+^ monoclonal antibody included in the ELISpot PRO Kit (Mabtech AB, Stockholm, Sweden) was used as an internal control at a dilution of 1:1000 as recommended by the manufacturer. Supplemented OPTmizer-CTS medium was used as a negative control.

### 2.5. Human IFNγ ELISpot Assay

The ELISPOT-IFNγ assay was performed using the ELISPOT-IFNγ PRO human IFNγ Kit 3420-ehst-10 (Mabtech AB, Stockholm, Sweden) as the antibody platform. In brief, 96-well plates pre-sensitized with human IFNγ antibody were washed 4 times with phosphate-buffered saline (PBS) (Lonza, Walkersville, MD, USA). The plates were then blocked with 200 μL of supplemented OPTmizer CTS medium per well for 30 min at RT. The medium was removed by inversion and 100 μL of the Spike, PHA, and anti-CD3^+^ stimuli were added to each well according to the plate design. Then, a different number of cells per well were added. Plates were incubated at 37 °C and 5% CO_2_ for 20 h.

After incubation, the cells were removed by inversion and the plates were washed 5 times with PBS; 100 μL per well of the detection monoclonal antibody anti IFNg (Mabtech AB, Stockholm, Sweden) diluted 1:200 in PBS with 0.5% FBS was added and plates were incubated for 2 h at RT, protected from light. Following incubation, the plates were washed 5 times with PBS and 100 μL of the tetramethylbenzidine (TMB, Mabtech AB, Stockholm, Sweden) substrate was added per well. The plates were incubated for 5 min protected from light, followed by 6 washes with deionized water. Finally, the plates were gently dried with a nonabrasive cellulose-fiber wiper and left to dry for 24–48 h protected from light at RT.

### 2.6. Data Acquisition

The ELISPOT-IFNγ plates were acquired 2 days after performing the assay using the CTL ImmunoSpot^®^ M6 automated analyzer (CTL, Cleveland, OH, USA) and later analyzed using the ImmunoSpot^®^ Software version 7.1.

After image acquisition, the spot-forming units (SFU) were counted using the automatically adjusted settings of the software (raw results), followed by the Quality Control function to eliminate artifacts. The counting parameters used were sensitivity 145, background balance 80, minimum spot size 0.00050 mm^3^, maximum spot size 9.6203 mm^3^, and spot separation 3. Absolute counting results were exported as individual Excel files for each plate.

### 2.7. Validation Protocol Design

For the validation of the ELISPOT-IFNγ analytic method, the linearity assay was performed using three replicates of increasing amounts of PBMCs (5 × 10^4^, 1 × 10^5^, 1.5 × 10^5^, 2 × 10^5^, 2.5 × 10^5^, 3 × 10^5^, and 3.5 × 10^5^ cells per well) from one of the volunteers. All experiments were performed in triplicate. After obtaining the results from this assay, we decided to use 2 × 10^5^ PBMCs for the rest of the experiments.

Intermediate precision, LOD, and LLOQ were evaluated through 10 replicates from each of the three donors for intermediate precision, and another operator assessed the same samples in the same conditions on a different day. The LLOQ was calculated according to the following equation [24]

LLOQ = mean + (3 × DS)

### 2.8. Statistical Analysis

The normality of the data was evaluated by using a Shapiro–Wilk test using the GraphPad Prism V9 software. To demonstrate the linearity of the method, we calculated the mean of the three determinations of SFUs in response to the Spike stimulus and their correlation with their corresponding cell density. The method was considered valid when it reached a multiple determination coefficient (r^2^) ≥ 0.93.

For repeatability and intermediate precision, we calculated the arithmetic mean and standard deviation (SD) of the 10 determinations of SFC in response to the Spike stimulus and then calculated the CV of each repetition of the three volunteers. The method was considered to be valid when it reached a cut-off CV ≤ 25 [25,26] in the overall results from the three volunteers. In the repeatability assay, data obtained from anti-CD3^+^ and PHA exposure were compared using a Student’s *t*-test. A value of *p* < 0.05 was considered statistically significant.

For the lower detection limit, we calculated the mean and standard deviation (SD) of the 10 determinations of SFC in response to the medium stimulus to obtain the CV of each result of the three volunteers.

## 3. Results

So far, there are no internationally accepted standardized operational procedures to validate the ELISPOT-IFNγ assay. Therefore, our laboratory required a validation design focused on the needs of our assay and the specific experimental conditions that needed to be validated. This design was based on the main parameters that contribute to the variability of the assay such as the LOD, LLOQ, linearity, repeatability, and intermediate precision. By validating these parameters, we could ensure that the results were reliable. The results were reported as spots produced by cells (2 × 10^5^/well).

To validate the ELISPOT-IFNγ, we considered parameters such as, linearity, LOD, LLOQ, and intermediate precision. An ELISPOT-IFNγ assay should be sensitive, precise, reproducible among operators, specific, and semi-quantitative. Additionally, it is required to show acceptable detection and quantification limits despite small variations in operating conditions.

### 3.1. Linearity

**Figure 1:**
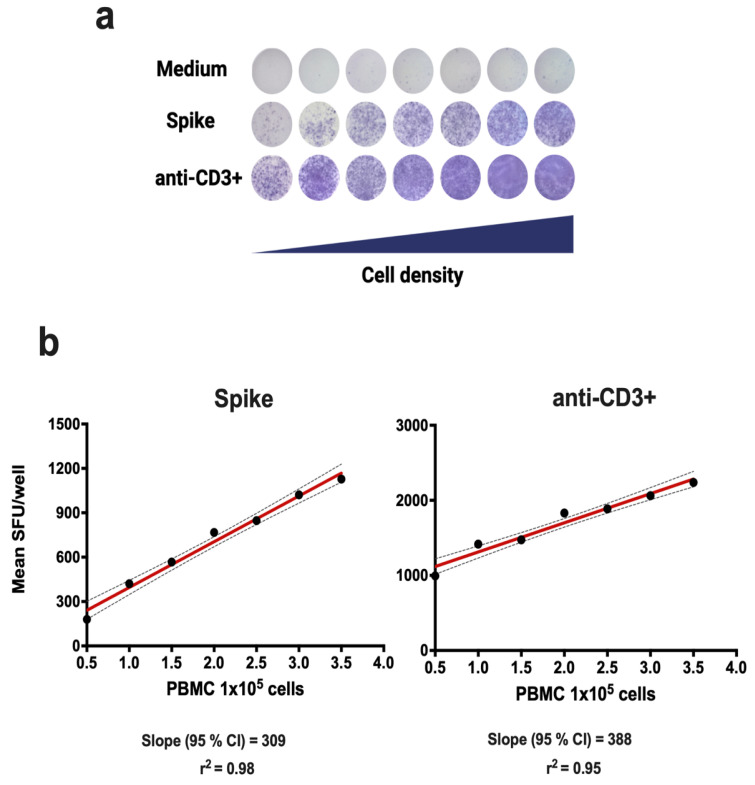
Linearity of the ELISPOT-IFNγ assay. The linearity of the method was evaluated in PBMCs from a single donor following 18 h of incubation with medium, Spike S1 protein, or anti-CD3^+^ antibody (positive control). (**a**) Scanned images of SFUs/well for PBMCs. The 95% confidence intervals (CIs) are shown in (**b**).

Linearity evaluates the ability of an assay to give results that are directly proportional to the concentration of the analyte being measured. In the case of ELISPOT-IFNγ, the number of IFNγ spots obtained should be directly proportional to the number of PBMCs placed in the wells. For the evaluation of linearity, PBMC from a single donor were used, evaluating nine replicates at seven different cellular densities (5 × 10^4^, 1 × 10^5^, 1.5 × 10^5^, 2 × 10^5^, 2.5 × 10^5^, 3 × 10^5^, and 3.5 × 10^5^ PBMC). The PBMC swere stimulated with the recombinant S1 protein (Table 1) and with anti-CD3^+^ antibody as positive control (Table 2). The results showed a significant linear relationship to produce IFNγ after stimulating the PBMC with the recombinant S1 protein (r^2^ = 0.98, *p* < 0.0001), or anti-CD3 antibody (r^2^ = 0.95, *p* < 0.001) as shown in Figure 1.

### 3.2. Limit of Detection (LOD)

**Figure 2:**
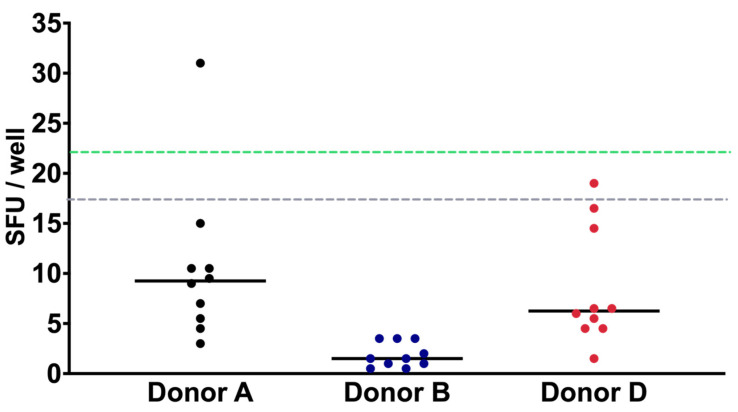
IFN-γ LOD and LLOQ characterization. The PBMC of three donors were cultured in OPTmizer medium and analyzed 10 times. Using the average of the results of each volunteer, the LOD of 17 spots/well (dotted gray line) and LLOQ of 22 spots/well (dotted green line) were calculated. Data are shown as the mean of the SFUs of each donor.

In ELISPOT-IFNγ, the LOD is defined as the minimum number of spots that can be detected in the unstimulated condition (OPTmizer medium). As ELISPOT-IFNγ is highly sensitive, we calculated the LOD by counting the non-specific background signals under non-stimulation conditions, i.e., the number of spots produced by cells in the OPTmizer medium. The PBMCs from three volunteers were analyzed, performing 10 replicates in duplicate (2 × 10^5^ cells/well). The immune response of volunteers accounted for the variability of the results obtained. The limit of detection was calculated according to Lee et al. [20] as:

LOD = O + 2SD(O)

where O is the average number of spots obtained in each well and SD is the standard deviation of the assay (Table 3). Using the LOD formula, the limits of detection was 26.51, 4.30, and 20.32 spots from donors A, B, and D, respectively. In this setting, the average of the detection limits of each volunteer resulted in a detection limit of 17 spots/well (Figure 2). In addition, the LLOQ was calculated by employing the results obtained from the LOD assay, corresponding to a value of 22 spots/well.

### 3.3. Repeatability

The repeatability of the analytical method was evaluated to demonstrate the accuracy of the assay. Repeatability indicates the variability in the results of successive measurements of the same sample and is usually expressed as the standard deviation or the CV. Measurements were performed in a standardized manner under the same operational conditions, including the same operator, equipment, laboratory, batch of reagents, and day.

**Figure 3:**
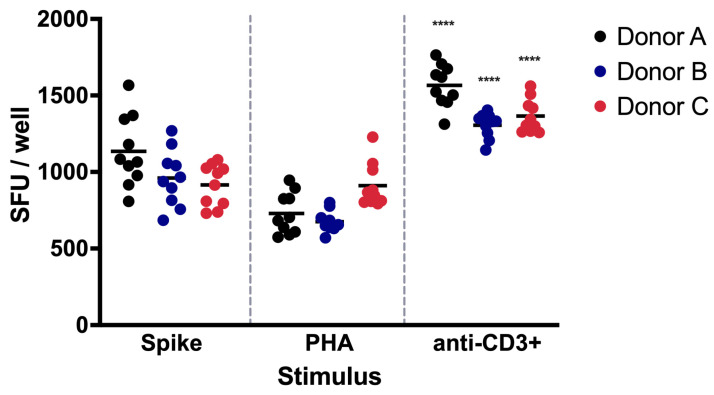
Repeatability assay of IFN-γ-producing PBMCs. The repeatability of the ELISPOT-IFNγ assay was evaluated by one analyst using PBMCs from three distinct donors. The PBMCs were exposed to Spike protein, PHA, or anti-CD3^+^ activating antibody. Data are shown as the mean of the SFUs. The significant differences in the SFUs after PHA or CD3^+^ exposure in each donor are indicated with asterisks (**** *p* < 0.0001).

For donor A, the coefficients of variation were 20.43, 18.41, and 8.89% for the Spike, PHA, and anti-CD3^+^ stimuli, respectively. In donor B, the coefficients of variation were 19.13, 10.21, and 17.73% for the Spike, PHA, and anti-CD3^+^ stimuli, respectively. Finally, in donor C, the coefficients of variation were 14.86, 15.40, and 8.00% for the same stimuli, respectively (Table 4, Figure 3).

### 3.4. Intermediate Precision

Intermediate precision indicates the reproducibility of the method when changes are applied to the analysis conditions. The intermediate precision of ELISPOT-IFNγ is a measure of the robustness and reliability of the method. This evaluation is fundamental to assessing the degree of uncertainty associated with the results and to ensure the comparability of the data obtained under different working conditions within the same laboratory. The intermediate precision was carried out in a standardized manner with two different operators and on different days. Three volunteers were analyzed, with 10 repetitions and each one in duplicate (2 × 10^5^ cells/well). The acceptance criterion for the CV was established at a value of less than or equal to 25% (CV ≤ 25%). The results represent the number of spots obtained from the analysis of three samples from donors stimulated with the Spike, PHA, and anti-CD3^+^ antibody. Intermediate precision contributes to ensuring data quality in terms of reproducibility.

From donor A, the coefficients of variation were 20.43, 18.41, and 8.89% for the Spike, PHA, and anti-CD3^+^ stimuli, respectively (Table 5). For donor B, the coefficients of variation were 19.13, 10.21, and 17.73% for the Spike, PHA, and anti-CD3^+^ stimuli, respectively (Table 6). In the case of donor C, the coefficients of variation were 14.86, 15.40, and 8.00% for the same stimuli, respectively (Table 7).

Intermediate precision evaluates the reproducibility of the method. In our results, we obtained a CV ≤ 21%, which indicates that the method is reliable and robust enough for application in biomedical research.

## 4. Discussion

ELISPOT-IFNγ is a functional assay used to evaluate the frequency of T cells that recognize a specific antigen. It is a semiquantitative and highly sensitive technique used to evaluate the immune response to infectious diseases, as well as the immune response induced by vaccines, including the SARS-CoV-2 vaccine [14,27]. However, the reproducibility and reliability of the results depend on factors such as the experience of the operators, especially of handling primary blood cells and reagents, as well as the instruments and equipment used [28].

ELISPOT-IFNγ is not only used for research or exploration purposes; it is widely employed in various preclinical or clinical studies, in which it has proven to be a useful tool for biomarker detection in diagnostics, immunotherapies, and vaccine evaluation [28,29].

Despite its extensive use in research and in clinical settings, ELISPOT-IFNγ does not have a regulatory guide available to develop and validate this type of assay. In addition, there are no appropriate reference standards or positive control samples that guarantee the certainty of the results. This creates the need to validate the method according to the objectives of each scientific protocol.

Since the ELISPOT-IFNγ assay must be sensitive, precise, reproducible, specific, and semi-quantitative, we assessed the suitability of the assay through these parameters.

Regarding the limit of detection, our results showed an LOD of 17 SFC (Table 1). The of LOD in our study was determined by counting non-specific background signals under non-stimulation conditions, using PBMCs from three volunteers. We performed 10 replicates in duplicate for each volunteer, analyzing 2 × 10^5^ cells per well. The LOD values for donors A, B, and C were 26.51, 4.30, and 20.32 spots, respectively, resulting in an average detection limit of 17 spots per well. The variation in the LOD between donors highlights individual differences in background immune activity, which is expected given the natural variability in immune responses across individuals. This variability is a crucial factor to account for, as it ensures the assay is sensitive enough to detect low levels of antigen-specific T cell responses, even in individuals with inherently higher baseline reactivity.

This LOD is lower than that reported by Schmittel et al. [29] suggesting that our technique is highly sensitive, suitable for evaluating low levels of cellular responses [17,18]. The obtained LOD was established as a negative control. Since the response of T cells can vary across healthy, sick, or immunosuppressed individuals [30,31], it is advisable to establish the LOD for the population to be studied.

Using the data obtained from the LOD calculations, we determined the Lower Limit of Quantification (LLOQ) to be 22 spots per well. In practical terms, the LLOQ ensures that even low-level immune responses to SARS-CoV-2 spike protein can be consistently measured, which is critical in assessing vaccine efficacy, particularly in populations with weaker immune responses such as the elderly or immunocompromised individuals.

Linearity indicates the number of cells that we should use for a reliable result [29,32]. In our linearity analysis, seven cell densities were tested, from 50,000 to 350,000 cells after stimulating the PBMCs with the S1 (Spike) protein and with the anti-CD3^+^ antibody. Correlation coefficients of r^2^ = 0.98 and r^2^ = 0.95 were obtained for the recombinant S1 protein and anti-CD3^+^, respectively, as shown in Figure 1. These results are in line with those reported by Waerlop [32], which confirms the accuracy of the procedure. The choice of the dilution factor is fundamental to ensure an adequate number of points for the calculation of other parameters.

Precision is defined as the proximity of values obtained across a series of measurements performed from the same sample under certain conditions [33]. We established the precision of the method through repeatability and intermediate precision tests. We performed repeatability tests with PBMC samples from three volunteers and 10 replicates. The intermediate precision tests were performed on different days and by different operators. The values obtained from repeatability tests showed a range of 675.25 to 1564.05 SFU, with a range from 579.25 to 2312.55 SFU for intermediate precision. The variation coefficients were ≤25%, both in the repeatability and intermediate precision tests, confirming the reliability of the results. These results demonstrate that the analytical method employing the ELISPOT-IFNγ assay is precise, since the criteria for evaluating repeatability and intermediate precision were met.

In our validation protocol, we incorporated the use of PHA, as an additional positive control, since it stimulates the production of IFNγ in PBMCs [23,34]. Surprisingly, lower levels of spots were detected after PHA stimulation when compared with the anti-CD3^+^ antibody, and significant differences were found when comparing the SFUs after PHA or CD3^+^ exposure in each donor (*p* < 0.0001).

The ELISPOT-IFNγ is a functional, highly quantitative assay with a wide range of detection limits. This makes the assay suitable for the study of antigen-specific cells and the evaluation of cellular responses to vaccines [35,36].

## 5. Conclusions

Cell-mediated memory responses play an important role in viral infections. In COVID-19, the T cell response to SARS-CoV-2 in patients and vaccinated subjects has been shown to contribute to disease and infection control [1]. The use of ELISPOT-IFNγ to study T lymphocyte responses in clinical trials has created the need for a proper validation method that supports its value in the development of new drugs and vaccines against infectious diseases.

## Figures and Tables

**Table 1:** Linearity of ELISPOT-IFNγ assay following exposure to Spike S1 protein.

Replicate	PBMC Density						
	5 × 10^4^	1 × 10^5^	1.5 × 10^5^	2 × 10^5^	2.5 × 10^5^	3 × 10^5^	3.5 × 10^5^
1	183	371	573	867	873	1054	925
2	201	448	495	654	969	998	1030
3	224	296	567	729	908	808	962
4	171	427	605	884	958	1214	1325
5	202	577	680	971	926	1190	1472
6	138	526	629	754	937	1142	1366
7	148	463	424	592	708	909	923
8	145	320	599	779	521	816	891
9	203	355	532	674	818	1061	1254
Mean SFU	179.44	420.33	567.11	767.11	846.77	1021.33	1127.55

PBMC: Peripherical blood mononuclear cells; SFU: spot-forming units; mean of duplicates.

**Table 2:** Linearity of the ELISPOT-IFNγ assay following anti-CD3^+^ stimulation.

Replicate	PBMC Density						
	5 × 10^4^	1 × 10^5^	1.5 × 10^5^	2 × 10^5^	2.5 × 10^5^	3 × 10^5^	3.5 × 10^5^
1	924	1444	1394	1744	1850	2327	2058
2	906	1399	1462	1668	1832	1863	2105
3	840	1508	1626	1908	2108	2010	1913
4	1098	1406	1413	1688	1739	2357	2616
5	1085	1505	1316	1743	1906	1840	2323
6	1019	1471	1542	2028	1804	1808	2650
7	1042	1322	1560	2341	2384	2873	2190
8	1048	1331	1437	1643	1715	1835	2183
9	978	1361	1521	1717	1638	1645	2102
Mean (SFU)	993.33	1416.33	1474.55	1831.11	1886.22	2062.00	2237.77

PBMC: Peripherical blood mononuclear cells; SFU: spot-forming units; mean of duplicates.

**Table 3:** LOD and LLOQ results of ELISPOT-IFNγ assay.

Replicate	Donor A	Donor B	Donor D
1	4.5	0.5	19
2	5.5	1	4.5
3	3	1.5	5.5
4	9	3.5	1.5
5	15	3.5	6
6	31	0.5	4.5
7	10.5	1	6.5
8	9.5	3.5	16.5
9	10.5	2	14.5
10	7	1.5	6.5
Mean (SFU)	10.55	1.85	8.5
SD (SFU)	7.981	1.226	5.911
LOD (SFU)	26.511	4.302	20.323
LLOQ (SFU)	34.49	5.52	26.23

SFU: spot-forming units.

**Table 4:** Summary of the repeatability of the ELISPOT-IFNγ assay performed by operator 1.

Replicate	Donor								
	A	B	C	A	B	C	A	B	C
	Spike			PHA			anti-CD3^+^		
1	1566.5	815	1024.5	825.5	684	1013	1634.5	1206	1417.5
2	1180	1055.5	1018.5	638	777	807.5	1502.5	1365	1561
3	1083	1269	1079.5	703.5	630.5	811.5	1674	1349	1299.5
4	1369.5	1183	1054.5	894.5	649	802.5	1764	1300.5	1262
5	1345	1041.5	991.5	824	570	792.5	1705.5	1368	1344
6	915.5	895.5	730	682	700	867	1456.5	1255	1266.5
7	1039.5	966	738	573.5	655	881	1620	1329.5	1304.5
8	1065.5	756.5	794	589.5	800	1227.5	1523.5	1332.5	1508
9	977	684.5	808.5	945.5	638	846	1467.5	1143.5	1258
10	808	937	914.5	608.5	649	1055	1312.5	1404	1433
Mean (SFU)	1134.95	960.35	915.35	728.45	675.25	910.35	1566.05	1305.3	1365.4
SD (SFU)	231.908	183.797	136.06	134.121	69.009	143.293	137.327	81.011	108.727
%CV	20.433	19.138	14.864	18.411	10.219	15.740	8.769	6.206	7.963

SFU: Spot-forming units; mean of duplicates SD: standard deviation; CV: Coefficient of variation.

**Table 5:** Intermediate precision in donor A.

Replicate	Operator 1 (Day 1)			Operator 2 (Day 2)		
	Spike	PHA	Anti-CD3^+^	Spike	PHA	anti-CD3^+^
1	1566.5	825.5	1634.5	1120.5	686.5	1672
2	1180	638	1502.5	1259	509.5	1595.5
3	1083	703.5	1674	1233	477	1592
4	1369.5	894.5	1764	1202.5	486.5	1617
5	1345	824	1705.5	1267.5	428.5	1656
6	915.5	682	1456.5	1244	616.5	1691.5
7	1039.5	573.5	1620	1087.5	562	1793
8	1065.5	589.5	1523.5	1131	708	1770.5
9	977	945.5	1447.5	1215.5	673	1662
10	808	608.5	1312.5	1041.5	645	1593.5
Mean (SFU)	1134.95	728.45	1564.05	1180.2	579.25	1664.3
SD (SFU)	231.909	134.122	139.057	79.055	99.736	71.435
%CV	20.433	18.412	8.891	6.698	17.218	4.292

SFU: Spot-forming units; mean of duplicates; SD: standard deviation; CV: coefficient of variation.

**Table 6:** Intermediate precision in donor B.

Replicate	Operator 1 (Day 1)			Operator 2 (Day 2)		
	Spike	PHA	Anti-CD3^+^	Spike	PHA	anti-CD3^+^
1	815	684	1206	1260	1099	1845.5
2	1055.5	777	1365	1285	973.5	1761
3	1269	630.5	1349	1238	1003.5	1670.5
4	1183	649	1300.5	1259.5	943.5	1716.5
5	1041.5	570	1368	1074	907.5	1735
6	895.5	700	1255	1091	1072	1784
7	966	655	1329.5	960	997	1842.5
8	756.5	800	1332.5	1097.5	1135	1692
9	684.5	638	643.5	1026	1000.5	1758
10	937	649	1404	885.5	903	1694.5
Mean (SFU)	960.35	675.25	1255.3	1117.65	1003.45	1749.95
SD (SFU)	183.797	69.009	222.577	138.661	78.153	60.671
%CV	19.138	10.219	17.730	12.406	7.788	3.467

SFU: Spot-forming units; mean of duplicates; SD: standard deviation; CV: coefficient of variation.

**Table 7:** Intermediate precision in donor C.

Replicate	Operator 1 (Day 1)			Operator 2 (Day 2)		
	Spike	PHA	Anti-CD3^+^	Spike	PHA	anti-CD3^+^
1	1024.5	1013	1417.5	811	1749	2608.5
2	1018.5	807.5	1561	882.5	1698.5	1692
3	1079.5	811.5	1299.5	923.5	1931	2141.5
4	1054.5	802.5	1262	919	1836.5	2201
5	991.5	792.5	1344	790	1751.5	2112.5
6	730	867	1266.5	671.5	1844.5	2114.5
7	738	881	1304.5	685.5	1803.5	2462.5
8	794	727.5	1508	822.5	2192	2461
9	808.5	846	1253	584	1697	2420.5
10	914.5	525	1433	907.5	2266.5	2910
Mean (SFU)	915.35	807.35	1364.9	799.7	1877	2312.4
SD (SFU)	136.06	124.332	109.286	117.747	199.54	334.454
%CV	14.864	15.4	8.006	14.723	10.63	14.463

SFU: Spot-forming units; mean of duplicates SD: standard deviation; CV: coefficient of variation.

## Data Availability

The data used to support the findings of this study are included within the article.

## References

[1] Miao G., Chen Z., Cao H., Wu W., Chu X., Liu H., Zhang L., Zhu H., Cai H., Lu X. (2023). From Immunogen to COVID-19 vaccines: Prospects for the post-pandemic era. Biomed. Pharmacother..

[2] Qian J., Zhang S., Wang F., Li J., Zhang J. (2024). What makes SARS-CoV-2 unique? Focusing on the spike protein. Cell Biol. Int..

[3] Jackson C.B., Farzan M., Chen B., Choe H. (2022). Mechanisms of SARS-CoV-2 entry into cells. Nat. Rev. Mol. Cell Biol..

[4] Plotkin S.A. (2010). Correlates of Protection Induced by Vaccination. Clin. Vaccine Immunol..

[5] Sherman A.C., Desjardins M., Baden L.R. (2022). Vaccine-Induced Severe Acute Respiratory Syndrome Coronavirus 2 Antibody Response and the Path to Acceleratisng Development (Determining a Correlate of Protection). Clin. Lab. Med..

[6] Chi W.-Y., Li Y.-D., Huang H.-C., Chan T.E.H., Chow S.-Y., Su J.-H., Ferrall L., Hung C.-F., Wu T.-C. (2022). COVID-19 vaccine update: Vaccine effectiveness, SARS-CoV-2 variants, boosters, adverse effects, and immune correlates of protection. J. Biomed. Sci..

[7] Liu L., Wang P., Nair M.S., Yu J., Rapp M., Wang Q., Luo Y., Chan J.F.-W., Sahi V., Figueroa A. (2020). Potent neutralizing antibodies against multiple epitopes on SARS-CoV-2 spike. Nature.

[8] Liu J., Chandrashekar A., Sellers D., Barrett J., Jacob-Dolan C., Lifton M., McMahan K., Sciacca M., VanWyk H., Wu C. (2022). Vaccines elicit highly conserved cellular immunity to SARS-CoV-2 Omicron. Nature.

[9] Yoon S.-W., Widyasari K., Jang J., Lee S., Kang T., Kim S. (2023). Kinetics of adaptive immune responses after administering mRNA-Based COVID-19 vaccination in individuals with and without prior SARS-CoV-2 infections. BMC Infect. Dis..

[10] Zhang Z., Mateus J., Coelho C.H., Dan J.M., Moderbacher C.R., Gálvez R.I., Cortes F.H., Grifoni A., Tarke A., Chang J. (2022). Humoral and cellular immune memory to four COVID-19 vaccines. Cell.

[11] Bercovici N., Duffour M.-T., Agrawal S., Salcedo M., Abastado J.-P. (2000). New Methods for Assessing T-Cell Responses. Clin. Diagn. Lab. Immunol..

[12] Leehan K.M., Koelsch K.A., Kurien B.T., Scofield R.H. (2015). T Cell ELISPOT: For the Identification of Specific Cytokine-Secreting T Cells. Western Blotting.

[13] Wang C., Yang S., Duan L., Du X., Tao J., Wang Y., Yang J., Lv Y., Li J., Zhang C. (2022). Adaptive immune responses and cytokine immune profiles in humans following prime and boost vaccination with the SARS-CoV-2 CoronaVac vaccine. Virol. J..

[14] Slota M., Lim J.-B., Dang Y., Disis M.L. (2011). ELISpot for measuring human immune responses to vaccines. Expert Rev. Vaccines.

[15] Streeck H., Frahm N., Walker B.D. (2009). The role of IFN-γ Elispot assay in HIV vaccine research. Nat. Protoc..

[16] Boaz M.J., Hayes P., Tarragona T., Seamons L., Cooper A., Birungi J., Kitandwe P., Semaganda A., Kaleebu P., Stevens G. (2009). Concordant Proficiency in Measurement of T-Cell Immunity in Human Immunodeficiency Virus Vaccine Clinical Trials by Peripheral Blood Mononuclear Cell and Enzyme-Linked Immunospot Assays in Laboratories from Three Continents. Clin. Vaccine Immunol..

[17] Wagenhäuser I., Almanzar G., Förg F.B., Stein A., Eiter I., Reusch J., Mees J., Herzog A., Vogel U., Frey A. (2024). Heterologous and homologous COVID-19 mRNA vaccination schemes for induction of basic immunity show similar immunogenicity regarding long-term spike-specific cellular immunity in healthcare workers. Vaccine.

[18] Konuma T., Hamatani-Asakura M., Nagai E., Adachi E., Kato S., Isobe M., Monna-Oiwa M., Takahashi S., Yotsuyanagi H., Nannya Y. (2024). Cellular and humoral immunogenicity against SARS-CoV-2 vaccination or infection is associated with the memory phenotype of T- and B-lymphocytes in adult allogeneic hematopoietic cell transplant recipients. Int. J. Hematol..

[19] Andreasson U., Perret-Liaudet A., Van Waalwijk Van Doorn L.J.C., Blennow K., Chiasserini D., Engelborghs S., Fladby T., Genc S., Kruse N., Kuiperij H.B. (2015). A Practical Guide to Immunoassay Method Validation. Front. Neurol..

[20] Lee J.W., Devanarayan V., Barrett Y.C., Weiner R., Allinson J., Fountain S., Keller S., Weinryb I., Green M., Duan L. (2006). Fit-for-Purpose Method Development and Validation for Successful Biomarker Measurement. Pharm. Res..

[21] Malyguine A.M., Strobl S., Dunham K., Shurin M.R., Sayers T.J. (2012). ELISPOT Assay for Monitoring Cytotoxic T Lymphocytes (CTL) Activity in Cancer Vaccine Clinical Trials. Cells.

[22] Carranza C., Juárez E., Torres M., Ellner J.J., Sada E., Schwander S.K. (2006). *Mycobacterium tuberculosis* Growth Control by Lung Macrophages and CD8 Cells from Patient Contacts. Am. J. Respir. Crit. Care Med..

[23] Vivekanandan M.M., Adankwah E., Aniagyei W., Acheampong I., Minadzi D., Yeboah A., Arthur J.F., Lamptey M., Abass M.K., Kumbel F. (2023). Impaired T-cell response to phytohemagglutinin (PHA) in tuberculosis patients is associated with high IL-6 plasma levels and normalizes early during anti-mycobacterial treatment. Infection.

[24] Pérez-Barrios Y., Aranguren-Mazorra Y., Zayas-Vignier C., Blain-Torres K., Hernández-Cedeño M., Rodríguez-Hernández Y., Álvarez-Tito M., Nuñez-Martínez D., González-Aznar E., Soubal-Mora J.P. (2018). Estandarización de un ELISA para la cuantificación de anticuerpos IgG contra vesículas de membrana externa de *Salmonella* Paratyphi A. Vaccimonitor.

[25] Corsaro B., Yang T., Murphy R., Sonderegger I., Exley A., Bertholet S., Dakappagari N., Dessy F., Garofolo F., Kierstead L. (2021). 2020 White Paper on Recent Issues in Bioanalysis: Vaccine Assay Validation, qPCR Assay Validation, QC for CAR-T Flow Cytometry, NAb Assay Harmonization and ELISpot Validation (Part 3 –Recommendations on Immunogenicity Assay Strategies, NAb Assays, Biosimilars and FDA/EMA Immunogenicity Guidance/Guideline, Gene & Cell Therapy and Vaccine Assays). Bioanalysis.

[26] Islam R., Vance J., Poirier M., Zimmer J., Khadang A., Williams D., Zemo J., Lester T., Fjording M., Hays A. (2022). Recommendations on ELISpot Assay Validation by the GCC. Bioanalysis.

[27] Deng Y., Li Y., Yang R., Tan W. (2021). SARS-CoV-2-specific T cell immunity to structural proteins in inactivated COVID-19 vaccine recipients. Cell Mol. Immunol..

[28] Janetzki S., Rueger M., Dillenbeck T. (2014). Stepping up ELISpot: Multi-Level Analysis in FluoroSpot Assays. Cells.

[29] Schmittel A., Keilholz U., Scheibenbogen C. (1997). Evaluation of the interferon-γ ELISPOT-assay for quantification of peptide specific T lymphocytes from peripheral blood. J. Immunol. Methods.

[30] Tary-Lehmann M., Hamm C.D., Lehmann P.V., Prabhakar U., Kelley M. (2008). Validating Reference Samples for Comparison in a Regulated ELISPOT Assay. Validation of Cell-Based Assays in the GLP Setting.

[31] Samri A., Durier C., Urrutia A., Sanchez I., Gahery-Segard H., Imbart S., Sinet M., Tartour E., Aboulker J.-P., Autran B. (2006). Evaluation of the Interlaboratory Concordance in Quantification of Human Immunodeficiency Virus-Specific T Cells with a Gamma Interferon Enzyme-Linked Immunospot Assay. Clin. Vaccine Immunol..

[32] Waerlop G., Leroux-Roels G., Lambe T., Bellamy D., Medaglini D., Pettini E., Cox R.J., Trieu M.-C., Davies R., Bredholt G. (2022). Harmonization and qualification of an IFN-γ Enzyme-Linked ImmunoSpot assay (ELISPOT) to measure influenza-specific cell-mediated immunity within the FLUCOP consortium. Front. Immunol..

[33] Körber N., Behrends U., Hapfelmeier A., Protzer U., Bauer T. (2016). Validation of an IFNγ/IL2 FluoroSpot assay for clinical trial monitoring. J. Transl. Med..

[34] Yang H., Sun J., Li Y., Duan W.-M., Bi J., Qu T. (2016). Human umbilical cord-derived mesenchymal stem cells suppress proliferation of PHA-activated lymphocytes in vitro by inducing CD4+CD25highCD45RA+ regulatory T cell production and modulating cytokine secretion. Cell. Immunol..

[35] Zhang W., Caspell R., Karulin A.Y., Ahmad M., Haicheur N., Abdelsalam A., Johannesen K., Vignard V., Dudzik P., Georgakopoulou K. (2009). ELISPOT assays provide reproducible results among different laboratories for T-cell immune monitoring--even in hands of ELISPOT-inexperienced investigators. J. Immunotoxicol..

[36] Ponce-de-León S., Torres M., Soto-Ramírez L.E., Calva J.J., Santillán-Doherty P., Carranza-Salazar D.E., Carreño J.M., Carranza C., Juárez E., Carreto-Binaghi L.E. (2023). Interim safety and immunogenicity results from an NDV-based COVID-19 vaccine phase I trial in Mexico. NPJ Vaccines.

